# Influx of multidrug-resistant organisms by country-to-country transfer of patients

**DOI:** 10.1186/s12879-015-1173-8

**Published:** 2015-10-28

**Authors:** Nico T. Mutters, Frank Günther, Anja Sander, Alexander Mischnik, Uwe Frank

**Affiliations:** Department of Infectious Diseases, Heidelberg University Hospital, Im Neuenheimer Feld 324, D-69120 Heidelberg, Germany; German Center for Infection Research, Heidelberg University Hospital, Heidelberg, Germany; Institute of Medical Biometry and Informatics, University of Heidelberg, Heidelberg, Germany

**Keywords:** Healthcare-associated infections, Infection control, Multidrug-resistant organisms, Carbapenemase

## Abstract

**Background:**

Multidrug-resistant organisms (MDRO) are a worldwide problem. International migration and travel facilitate the spread of MDRO. Therefore the goal of our study was to assess the risk of influx of MDRO from patients transferred to one of Central Europe’s largest hospitals from abroad.

**Methods:**

A mono-centre study was conducted. All patients transferred from other countries were screened; additional data was collected on comorbidities, etc. Presence of carbapenemases of multidrug-resistant Gram-negatives was confirmed by PCR. The association between length of stay, being colonized and/or infected by a MDRO, country of origin, diagnosis and other factors was assessed by binomial regression analyses.

**Results:**

From 2012 to 2013, one fifth of all patients were colonized with MDRO (Methicillin-resistant Staphylococcus aureus [4.1 %], Vancomycin-resistant Enterococci [2.9 %], multidrug-resistant Gram-negatives [12.8 %] and extensively drug-resistant Gram-negatives [3.4 %]). The Gram-negatives carried a variety of carbapenemases including OXA, VIM, KPC and NDM. The length of stay was significantly prolonged by 77.2 % in patients colonized with a MDRO, compared to those not colonized (*p*<0.0001).

**Conclusions:**

Country-to-Country transfer of patients to European hospitals represents a high risk of introduction of MDRO and infection control specialists should endorse containment and screening measures.

## Background

Multidrug-resistant organisms (MDRO) are a worldwide problem and the speed at which resistance rates are increasing is worrying. Antibiotic resistance crosses international boundaries and spreads easily between continents [1]. Surveillance systems such as the European Antimicrobial Resistance Surveillance System network report a continuing increase in antimicrobial resistance. International travel and patient transfers from hospitals located in high-prevalence regions to hospitals in low-prevalence regions facilitate the spread of MDRO. This condition triggers the introduction and establishment of MDRO into previously unaffected or less affected regions [2]. Methicillin-resistant *Staphylococcus aureus* (MRSA), for example, is a major cause of healthcare- and community associated infections worldwide [3]. It appears, however, to be regionally very differently distributed. While prevalence rates in some countries such as the Netherlands or Scandinavian countries are very low, MRSA are highly prevalent in the USA, Japan and Greece [4–6]. In addition, the epidemiology of vancomycin-resistant *Enterococci* (VRE) exhibits a remarkable geographic diversity and variable temporary trends [6–9]. Though the situation in Gram-positives appears to be rather stable, the situation in multidrug-resistant Gram-negatives (MDR-GN) is disquieting. The extensive use of broad-spectrum antibiotics has triggered the proliferation of highly resistant Gram-negative organisms [10]. In the last decade these organisms have spread over the whole world and can be found (sporadically or endemic) almost everywhere now [11–13].

The identification of patients carrying these MDRO is of utmost importance for infection control. Screening and pre-emptive contact isolation in single rooms, however, is also very costly. In addition, being colonized might also increase the risk of infection and, hence, has an influence on morbidity and mortality as well. This could result in an increased length of stay (LOS) and higher in-hospital costs. We therefore conducted a study to estimate the risk of influx of MDRO from country-to-country transfer of patients.

## Methods

### Study population

Only patients transferred from abroad to Heidelberg University Hospital (HUH) were included. HUH is a one of Germany’s largest hospitals with 2,000 beds and 90,000 in-patients per year. HUH provides a full range of medical and surgical services, including transplantation programmes, and is one of the leading medical centres in Europe. Approximately 500 patients per year are transferred from other countries to HUH, mainly from the Middle East.

Start of the study was the 15^th^ of July 2012; endpoint was the 15^th^ of September 2013. Patients were included and screened if they spent 48 h or longer in a hospital abroad (i.e. outside of Germany) within 14 days prior to admission to HUH and if they had an expected stay of 24 h or longer at HUH. All patients were pre-emptively isolated in single rooms until negative screening results were obtained. The screening regimen included nasal, rectal and if applicable wound or stoma swabs (eSwab, Copan). In addition, clinical specimens were also checked for MDRO. Infection with an MDRO was defined as patients with clinical MDRO infection. Criteria were based on CDC/NHSN definitions for healthcare-assocoiated infections (http://www.cdc.gov/HAI/infectionTypes.html). We retrospectively collected additional data including: Patients’ age and gender, country of origin and/or residency, diagnosis related groups (DRG), type of hospital ward, LOS, type of specimen sent for microbiological testing, and type of pathogen isolated.

### Microbiological methods

Swabs were inoculated on Columbia 5 % sheep blood agar plate (BD) and chromogenic plates for MRSA detection (ChromAgar MRSA II, BD), for ESBL/MDR-GN detection (chromID ESBL, bioMerieux), and for VRE detection (Chromagar VRE, Mast Diagnostica); incubated aerobically for 48 h at 36 °C. Columbia-sheep blood agar was used as growth control. If growth on chromogenic plates was detected, identification by matrix-assisted laser desorption ionization-time-of-flight mass spectrometry (Bruker, Germany), as described elsewhere, was performed [14]. Susceptibility-testing was performed by VITEK2 (bioMerieux) according to EUCAST criteria. Presence of resistence genes was confirmed by multiplex RT-PCR; mecA, femB for MRSA, vanA, vanB for VRE, and various carbapenemase resistance genes for MDR-GN as described elsewhere [15].

### Classification

MDR-GN were classified as multidrug-resistant (MDR) and extensively drug-resistant (XDR) as described elsewhere [16].

### *Spa*-typing

*Spa* typing was performed on each of the MRSA isolates as described elsewhere [17].

### Statistics

For descriptive purposes, arithmetic mean value, standard deviation, median, interquartile range, and cumulative frequencies were calculated as appropriate. The LOS follows a zero inflated distribution. Therefore we performed negative binomial regression to assess the influence of colonization with MDRO and other factors on LOS. Two different models were fitted to patients with information on diagnosis (*n* = 343). The first model contained being colonized, age and main diagnosis of the patient. The diagnoses were included due to the obvious influence of severity of underlying disease on the LOS. The second model contained being colonized or infected with VRE, MRSA, MDR-GN or XDR-GN, age, and main diagnosis. To evaluate possible risk factors of being colonized logistic regression analysis was performed, including the country areas of Africa, Asia, the Middle East, Non-EU Europe and EU-Europe. The model included age, grouped DRG diagnoses, and the aforementioned country areas. *P* values of ≤0.05 were regarded as statistically significant. Statistical analysis was performed using SAS Software (Version 9.2, SAS, Inc., Cary, NC, USA).

### Ethical considerations

At HUH, all MDRO strains are routinely collected for disease surveillance and outbreak detection. The current study thus is descriptive of a bacterial collection and microbiological characteristics could only be combined with the sex, age, clinical outcome, hospitalization, and travel history for the patients from which the strains were isolated. Additionally, data collected from patients with negative screening results was anonymised and restricted to sex, age, clinical outcome, hospitalization, and travel history. Ethical approval was therefore not required. Also, the German Act relating to the control of communicable diseases obliges HUH to monitor the MDRO on a regular basis (Infektionsschutzgesetz; http://www.gesetze-im-internet.de/ifsg/). For these reasons, consent to analyze the bacterial samples for this research project was not obtained from the patients.

## Results

### Demographic data

The mean age of all screened patients was 42.0 years (±23.1 SD); 59.9 % were males. The majority came from the Middle East (57.5 %), followed by Non-EU-Europe (17.9 %), EU-Europe (13.0 %), and Africa (8.5 %) (Table 1). Of all countries, patients most frequently came from Saudi Arabia (15.9 %), Russia (13.5 %), and Kuwait (10.1 %). Most common diagnoses at admission were solid organ malignancies (43.4 %), hereditary genetic disorders (9.9 %), acute (9.0 %) and chronic (8.5 %) internal medical diseases, and infections (7.6 %).

**Table 1:** Country of origin of screened patients

Area code	Number / Percent
Middle East	238 / 57.5
Non-EU-Europe	74 / 17.9
EU	54 / 13.0
Africa	35 / 8.5
Asia	10 / 2.4
Americas^a^	2 / 0.5
Australia^a^	1 / 0.2
Total	414

EU (including EFTA [European Free Trade Association] countries Switzerland & Norway); Non-EU-Europe (including Russia)

^a^:excluded from the logistic regression analysis

### Microbiological results

Eighty-seven of 414 patients were colonized with MDRO (MRSA [4.1 %], VRE [2.9 %] or MDR-GN [12.8 %] and XDR-GN [3.4 %]. The majority (83.9 %, *n* = 73/87) was colonized with only one bacterial species, while 16.1 % (*n* = 14/87) were colonized with either two or more different MDRO. Of all isolated Gram-negatives, 81.1 % (*n* = 60/74) were MDR-GN, and 18.9 % XDR-GN, respectively. The XDR-GN carried various carbapenemases from different classes. Detailed information on the pathogens is listed in table 2. One third (26/87; 29.9 %) of the patients was diagnosed with an infection caused by a MDRO upon admission. The most frequent infections were wound infections and urinary tract infections, accounting for 38.5 % (*n* = 10/26) and 38.5 % (*n* = 10/26), respectively; followed by intra-abdominal infections 19.2 % (5/26) and catheter related infections 3.8 % (1/26). The most frequent MDRO causing infections were 53.8 % MDR-GN (*n* = 14/26), 19.2 % XDR-GN (*n* = 5/26), 19.2 % MRSA (*n* = 5/26), and 7.7 % VRE (*n* = 2/26). The type of swabs obtained for specimen screening were 48.0 % nasal, 45.8 % rectal, 5.0 % wound and 1.2 % stoma.

**Table 2:** Distribution of multidrug–resistance among isolates of colonized patients

Species	No. (%) of isolates	No. (%) of MDR–GN	No. (%) XDR–GN	No. and type of Carbapenemase in XDR–GN
*Escherichia coli*	42 (40.4)	39 (65)	3 (21.4)	1 none
				1 NDM–1
				1 KPC
MRSA	18 (17.3)	N/A	N/A	N/A
* Klebsiella pneumoniae*	18 (17.3)	14 (23.3)	4 (28.6)	2 none
				1 NDM–1
				1 OXA–48
VRE (all *E. faecium*)	12 (11.5)	N/A	N/A	N/A
* Pseudomonas aeruginosa*	6 (5.8)	3 (5)	3 (21.4)	2 none
				1 VIM–1
*Acinetobacter baumanii*	3 (2.9)	0 (0)	3 (21.4)	3 OXA–23
* Enterobacter cloacae*	3 (2.9)	2 (3.3)	1 (7.1)	1 VIM–4
* Proteus mirabilis*	1 (1.0)	1 (1.7)	0 (0)	N/A
* Klebsiella oxytoca*	1 (1.0)	1 (1.7)	0 (0)	N/A
total	104 (100)	60 (100)	14 (100)	9

*N/A* not applicable, *MRSA* methicillin–resistant *Staphylococcus aureus*, *VRE* vancomycin–resistant Enterococci, *NDM–1* New Delhi metallo–beta–lactamase 1, *KPC Klebsiella pneumoniae* carbapenemase, *OXA–23/48* oxacillinase group beta–lactamase 23/48, *VIM–1/4* Verona integron–encoded metallo–beta–lactamase 1/4

### *Spa* typing

In total, *spa* typing resolved 12 distinct *spa* types for the collected MRSA strains (t002, t003, t024, t030, t037, t044, t127, t304, t502, t688, t3343, t3379). One patient was colonized with MRSA strains of three different *spa* types (t127, t304, t3343). Two of the MRSA isolates belonged to the clonal complex (CC) 5, which includes the Rhine-Hesse MRSA prototype and is prevalent in the area where HUH is located. Additionally, two MRSA strains belonged to the *spa* type t044, representing the major European cMRSA clone ST80. The majority of the collected MRSA-isolates (83 %) belonged to low prevalence *spa* types with local prevalence rates below 1.5 %.

### Regression analysis

#### Influence of colonization status on length of stay

The median LOS for all patients was 10 days (inter quartile range (IQR) = 3–20), and 14 days (IQR: 6–23) when including patients with underlying diseases as classified in DRG registry. LOS was prolonged by 77.2 % in colonized patients (Incidence rate ratio (IRR) = 1.78, p < 0.001) compared to uncolonized patients adjusting for diagnose groups and age. Colonized trauma patients had the longest prolongation of LOS compared to all other colonized patients (125.0 %, IRR = 2.25, *p* = 0.033). VRE or MDR-GN positive patients were associated with statistically significantly longer LOS (VRE: IRR = 2.9, p<0.001; MDR-GN: IRR = 1.7, *p*<0.001) (Table 3). The models were run including patients that had more than one isolate, since excluding them did not change the parameter estimates, nor improve the model fit.

**Table 3:** Influence of colonization status on LOS (Model 1) and Influence of specific MDRO on LOS (Model 2)

	Model 1			Model 2		
Parameter	IRR	95 % CI	*P* value	IRR	95 % CI	*P* value
Colonized	1.772	[1.324; 2.371]	<0.001	–	–	–
Colonized trauma patients	2.250	[1.069;4.735]	0.033	–	–	–
VRE	–	–	–	2.882	[1.573; 5.280]	<0.001
MRSA	–	–	–	1.343	[0.801; 2.252]	0.264
MDR–GN	–	–	–	1.738	[1.254; 2.411]	<0.001
XDR–GN	–	–	–	1.338	[0.700; 2.561]	0.379
Age	0.995	[0.990; 1.001]	0.122	0.994	[0.988; 1.000]	0.052
Diagnosis related groups ^a^						
Surgical diseases	1.573	[0.702; 3.526]	0.271	1.122	[0.519; 2.424]	0.771
Mild internal diseases	0.499	[0.274; 0.909]	0.023	0.370	[0.213; 0.643]	<0.001
Internal medical diseases (all others)	0.727	[0.404; 1.310]	0.2891	0.523	[0.308; 0.890]	0.017
Hereditary genetic disorders	0.920	[0.493; 1.718]	0.794	0.629	[0.354; 1.118]	0.114
Malignancies	0.945	[0.578; 1.547]	0.823	0.679	[0.443; 1.041]	0.076
Infection	1.079	[0.582; 2.000]	0.810	0.783	[0.446; 1.376]	0.396

^a^Trauma defined as reference; *IRR* incidence rate ratio, *CI* confidence interval

### Influence of region of origin on colonization status

Of all 414 patients included in the study, 3 were excluded from the logistic regression analysis since patients from the Americas and Australia were all uncolonized and their numbers were too low to be included. The model was adjusted for diagnose groups and age. Patients being transferred from Africa (odds ratio [OR]: 2.5, 95 % confidence interval [CI]: 0.8-7.9, *p* < 0.12) and the Middle East (OR: 2.4, 95 % CI: 1.0-5.7, *p*<0.06) had the highest risk of being colonized with a MDRO, followed by patients from Non-EU countries (OR: 1.4, 95 % CI: 0.5-4.1, *p* < 0.52). Patients transferred from Asia (OR: 0.8, 95 % CI: 0.1-7.9, *p*<0.9) on the other hand, had a lower risk of being colonized with an MDRO compared to patients from the EU. However, the results did not reach statistical significance (Table 4).

**Table 4:** Influence of region of origin on colonization status

Effect	Odds ratio	95 % CI	*P* value
Country Area (Reference: EU)			
Africa	2.493	[0.785; 7.914 ]	0.121
Asia	0.833	[0.088; 7.869]	0.873
Middle East	2.366	[0.980; 5.715]	0.056
Non-EU-Europe	1.411	[0.489; 4.077]	0.524
Age	1.012	[0.999; 1.025]	0.064
Diagnosis related groups ^a^			
Surgical diseases	0.938	[0.100; 8.799]	0.956
Mild internal diseases	1.758	[0.500; 6.184]	0.379
Internal medical diseases (all others)	1.220	[0.322; 4.619]	0.769
Hereditary genetic disorders	1.737	[0.435; 6.925]	0.434
Malignancies	2.856	[1.200; 6.798]	0.017
Infection	2.709	[0.820; 8.944]	0.102
Trauma	6.067	[2.108; 17.458]	<0.001

^a^No diagnosis (none) defined as reference; CI: confidence interval

## Discussion

We screened all patients transferred from abroad for the presence of MDRO and collected additional clinical and demographic data to assess the impact of MDRO colonization and infection on the individual and the risk of influx of MDRO into large university hospitals. The profile of HUH is representative of other large university hospitals. The analyzed data show that a considerable proportion of patients transferred from abroad are colonized with MDRO, mainly with MDR- and XDR-GN. The proportion of colonization, compared to patients from other hospitals in Germany, was equal with regards to VRE (2.9 % vs. 2.9 %), while it was superior with regards to all other MDROs [MRSA (4.1 % vs. 2.4 %), MDR-GN (12.8 % vs. 2.2 %), XDR-GN (3.4 % vs. 0.3 %)].

Of all Gram-negatives 18.9 % were XDR-GN (*n* = 14/74) carrying various carbapenemases of different classes including OXA-48, VIM-1, KPC and NDM-1 (Table 2). The carbapenemases carrying strains were mainly Enterobacteriaceae, such as *E. coli* and *K. pneumoniae*, which are the main reservoir species for NDM, KPC, and OXA-48 carbapenemases worldwide [13]. Patients carrying XDR-GNs repeatedly act as index patients, leading to outbreaks when transferred from high prevalence areas [18, 19]. In addition, infections with MDR-GN or XDR-GN are associated with worse clinical outcome and prolonged LOS [20–22]. Not only infections, but also colonizations with MDRO can have an impact on clinical outcome and can result in prolonged LOS [20, 23]. In some patients, being colonized also increases the risk of developing an infection with the same MDRO [24, 25]. Our results are concordant with these findings. Being infected or colonized with a MDRO increased the LOS significantly and considerably prolonged the duration of the hospital stay. The most substantial and statistically significant increase in LOS was due to MDR-GN and VRE. The prevalence of VRE in our study, however, was lowest among all MDRO and the overall impact of MDR-GN was therefore much higher. Especially E. coli, with an incidence more than twice as high as any other isolated microorganism in this study, had a major impact. The advantage of our study compared to others investigating the impact of colonization of MDRO on LOS [26], is our adjustment for the severity of underlying illnesses.

The present study also has some minor limitations. Our results might overestimate the prevalence rates of MDRO in the different regions, since patients transferred to a university hospital are usually sicker than those treated in smaller regional hospitals. In addition, our study population consisted of patients who previously had contact with hospitals in their home country, thus having an intrinsic increased risk of being colonized with a MDRO. As we only included patients transferred from abroad, we cannot entirely exclude the, however unlikely, possibility that those patients had been to German hospitals in earlier years. Nevertheless, these are exactly the kind of patients that are transferred to large University Hospitals from abroad and probably reflect the prevalence rates that infection control specialists in Western countries have to expect. The external validity of our results might only be hampered by the fact that 57.5 % of all patients came from the Middle East. Thus, results might differ in hospitals where the majority of foreign patients come from other areas.

The low MRSA rates in our study, however, might be influenced by the fact that most of the included patients came from the Middle East and Non-EU Europe and only few came from Asia or the Americas. In fact, the highest MRSA rates worldwide are found in North America and Japan [6]. *Spa* typing resolved that our collected MRSA strains consisted of highly diverse *spa* types and only four of all MRSA positive patients had *spa* types (CC5 and ST-80) also present in the local endemic lineages. Thus, the majority of MRSA strains from international patients are highly diverse and not usually found in the local European environment.

## Conclusions

Our data show that a considerable proportion of transferred patients are colonized with MDRO, mainly with MDR- and XDR-GN, which carry a variety of different carbapenemases. Infections with MDRO occurred in approximately one-third of all colonized patients. LOS was significantly prolonged in all colonized patients. In summary, country-to-country transfer of patients to large university hospitals represents a risk of introduction of MDRO. We would like to emphasize that infection control specialists should maintain vigilance, endorse containment and screening measures, and receive transferred patients in an area of the hospital equipped to manage isolation for MDRO [2].

## Abbreviations

MDRO: Multidrug-resistant organisms
MRSA: Methicillin-resistant Staphylococcus aureus
VRE: Vancomycin-resistant Enterococci
MDR-GN: Multidrug-resistant Gram-negatives
XDR-GN: Extensively drug-resistant Gram-negatives
LOS: Length of stay
HUH: Heidelberg University Hospital
DRG: Diagnosis related groups
IQR: Inter quartile range
IRR: Incidence rate ratio
CI: Confidence interval
OR: Odds ratio
